# *Phox2b* Influences the Development of a Caudal Dopaminergic Subset

**DOI:** 10.1371/journal.pone.0052118

**Published:** 2012-12-14

**Authors:** Elisa J. Hoekstra, Lars von Oerthel, Annemarie J. A. van der Linden, Marten P. Smidt

**Affiliations:** 1 Neuroscience and Pharmacology, Rudolf Magnus Institute of Neuroscience, University Medical Center Utrecht, Utrecht, The Netherlands; 2 Center for Neuroscience, Swammerdam Institute for Life Sciences, University of Amsterdam, Amsterdam, The Netherlands; Baylor College of Medicine, Jiao Tong University School of Medicine, United States of America

## Abstract

The developing mesodiencephalic dopaminergic (mdDA) neuronal field can be subdivided into several molecularly distinct domains that arise due to spatiotemporally distinct origins of the neurons and distinct transcriptional pathways controlling these neuronal subsets. Two large anatomically and functionally different subdomains are formed that eventually give rise to the SNc and VTA, but more subsets exist which require detailed characterization in order to better understand the development of the functionally different mdDA subsets, and subset-specific vulnerability. In this study, we aimed to characterize the role of transcription factor *Phox2b* in the development of mdDA neurons. We provide evidence that *Phox2b* is co-expressed with TH in a dorsal-caudal subset of neurons in the mdDA neuronal field during embryonic development. Moreover, *Phox2b* transcripts were identified in FAC-sorted *Pitx3* positive neurons. Subsequent analysis of *Phox2b* mutant embryos revealed that in the absence of *Phox2b*, a decrease of TH expression occurred specifically in the midbrain neuronal subset that normally co-expresses *Phox2b* with TH. Our data suggest that *Phox2b* is, next to the known role in the development of the oculomotor complex, involved in the development of a specific caudal mdDA neuronal subset.

## Introduction

In Parkinson's disease (PD) the onset is highlighted by a specific degeneration of meso-diencephalic dopaminergic (mdDA) neurons of the Substantia nigra pars compacta (SNc). To understand this neuron specific vulnerability, a thorough understanding of the development of mdDA neurons is essential. Based on a study in *Lmx1b* null mutants, it was suggested that *Lmx1b* is required for the generation of properly differentiated mdDA neurons. This study demonstrated a remarkable loss of developing mdDA neurons co-expressing *Pitx3* and *Th*, in E12.5 *Lmx1b*−/− tissue [1]. Furthermore, *Lmx1b* plays an important role in the correct specification of the mid-hindbrain boundary (MHB), where it regulates expression of *Fgf8, Wnt1* and several isthmus-related transcription factors, and it is required for the inductive activity of the isthmic organizer (IsO) itself [2], [3]. Notably, the clear reduction of mdDA neurons in *Lmx1b*−/− embryos is likely due to an early loss of a large part of the midbrain, due to affected MHB patterning [2].

Previously, we identified *Lmx1b* as upstream activator of *Phox2a* (unpublished data). These data were recently confirmed as they showed the dependence of *Phox2a* expression in oculomotor neurons on the activity of *Lmx1b* [4]. *Phox2a* is expressed in the midbrain oculomotor complex (OMC), that partially overlaps with the mdDA neuronal field during development. Over the years, *Phox2a* has been identified as an important regulator of midbrain motorneuron development, in mice and humans, and in *Phox2a*−/− mice, midbrain oculomotor neurons are absent [5]–[7]. A recent study in chick suggested that exogenous *Phox2a* can induce a complete OMC molecular program, and can act as a primary developmental determinant for the oculomotor complex [8]. The functional paralogue of *Phox2a*, *Phox2b*, is expressed in the hindbrain where it plays an essential role in the specification of cranial motor neurons [6], [9]–[11]. Importantly, *Phox2b* is expressed in the OMC as well, and recently some degree of cooperation between *Phox2a* and *Phox2b* was discovered in motorneuron development [12]. In addition, molecular evidence was provided that *Phox2b* can regulate the expression of *Phox2a* by specifically interacting with the 5′-regulatory region of *Phox2a* [13].

The collective data on regulation of *Phox2a* by *Lmx1b* and *Phox2b*, aimed our interest towards a putative role of *Phox2b* in caudal subset specification of mdDA neurons. In this study, we show that *Phox2b* is co-expressed with TH and importantly, with *Pitx3* in mdDA neurons during development. Interestingly, subsequent analysis of *Phox2b* null mutants revealed decreased expression of TH in the exact subset that normally expresses *Phox2b*, indicating that *Phox2b* plays a role in the development of this specific caudal subset of mdDA neurons.

**Figure 1:**
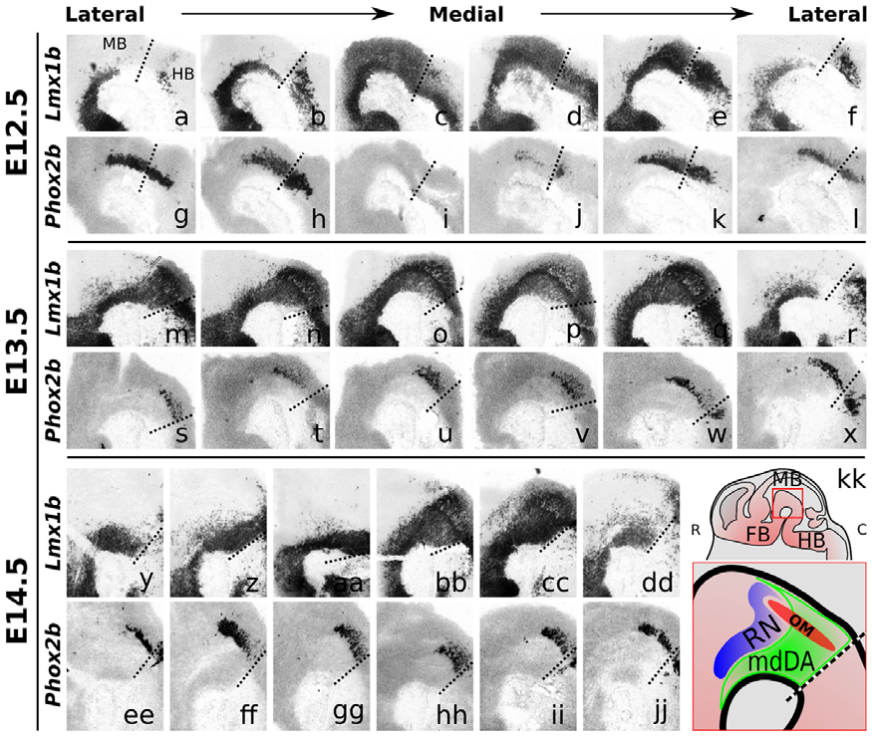
Expression of *Phox2b* and *Lmx1b* in the midbrain of wild-type mouse embryos. Sagittal sections of E12.5, E13.5 and E14.5 wild-type mouse brains, from lateral to medial to lateral. ISH staining is shown for *Phox2b*, and for *Lmx1b* as a reference. Dashed lines represent the mid-hindbrain boundary. (**a–l)** *Lmx1b* and *Phox2b* mRNA expression at E12.5. *Lmx1b* is broadly expressed throughout the midbrain, in P1, P2 and P3, and in the hindbrain. Except for the most medial part, *Phox2b* is expressed in the posterior midbrain and anterior hindbrain. (**m–x**) *Lmx1b* and *Phox2b* mRNA expression at E13.5, and (**y–jj**) at E14.5. (**kk**) Schematic overview of a sagittal mouse brain at E14.5, depicting several neuronal fields in the midbrain area (red box). *C, caudal; R, rostral; FB, forebrain; MB, midbrain; HB, hindbrain; RN, red nucleus; OM, oculomotor complex*.

## Materials and Methods

### Ethics statement

Mice were bred in our laboratory under standard conditions and all procedures were fully approved by the Dutch Ethical Committee (DEC) for animal experimentation of the University Medical Center Utrecht in the Netherlands (DEC-UMC-U) and international guidelines.

### Animals

Experiments were carried out in C57Bl/6J wild-type mice (Charles River). Pregnant mice were decapitated or euthanized by CO2 asphyxiation and embryos were collected at E12.5, E13.5 and E14.5 (the morning of detection of a copulatory plug was considered E0.5). Pups were euthanized by CO2 asphyxiation and brains were isolated at postnatal (P) day 0, P7 and P14. *Phox2b-LacZ* mutant mice [14] were maintained under the same conditions. Embryos were collected at E12.5, E14.5, E16.5 and E18.5. Since homozygous mutants rarely survive after E13.5, due to a noradrenalin deficit [10], we treated drinking water of pregnant *Phox2b-LacZ/+* mice by supplementing with 100 ug/mL of L-phenylephrine (Merck), 100 ug/mL isoproterenol (Sigma) and 2 mg/mL ascorbic acid (Sigma), from E8.5 onwards. Animals were genotyped by means of PCR, using a forward primer located in intron 1 of the *Phox2b* coding sequence (5′-GTTCTGGTTCAGTGGCCCTTC) and a reverse primer in the *LacZ* inserted sequence (5′-AGGCTGCGCAACTGTTGG) resulting in a product of 260 bp in mutants, or no product, in wild-type animals. To discriminate between heterozygous and homozygous *LacZ* mutants, the same forward primer was used, with a reverse primer in the wild-type sequence directly after the LacZ insertion (5′-GCAAACGAATCACGCAATTAAG). *Pitx3-GFP* mice were described previously [15]. RNA from *Pitx3-GFP/+* embryos was used for fluorescence-activated cell sorting (FACS).

**Figure 2:**
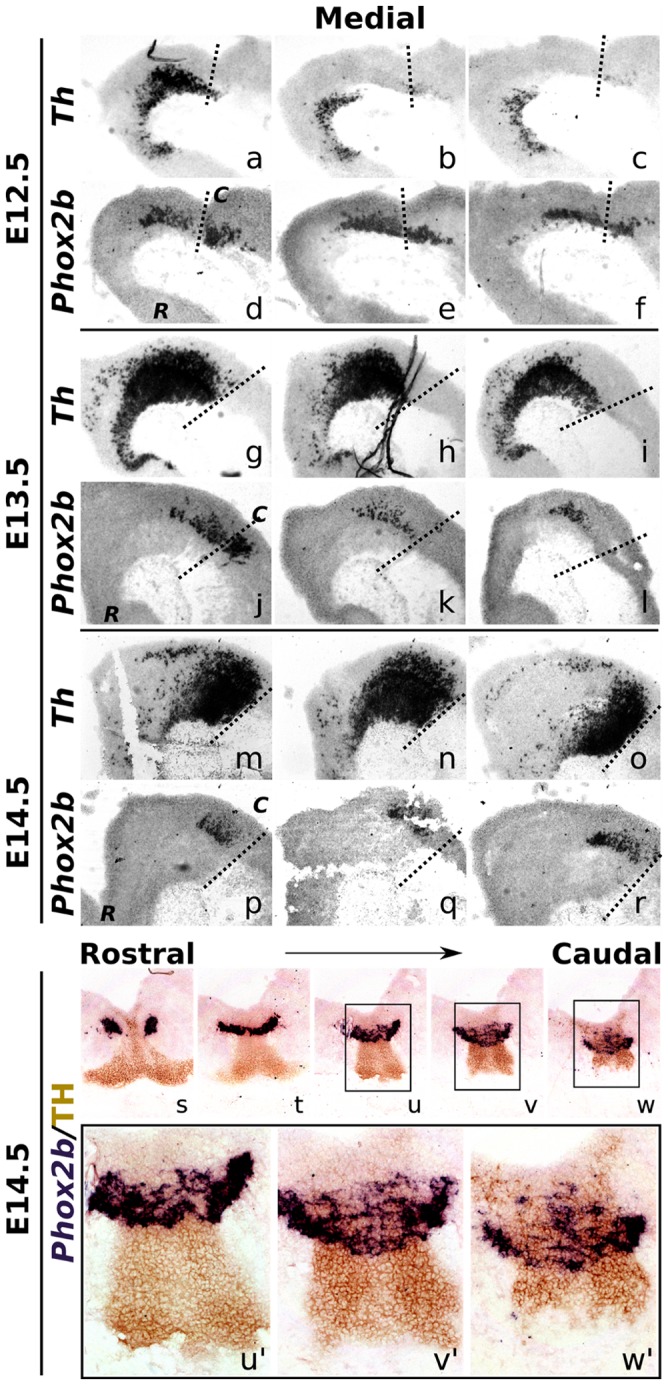
*Phox2b* expression overlaps with *Th* during development. (**a–f**) *Th* and *Phox2b* mRNA expression at E12.5 wild-type mouse brains from medial to lateral, (**g–l**) at E13.5 and (**m–r**) at E14.5. *Th* marks the mdDA neuronal field. Dashed lines represent the mid-hindbrain boundary. *Phox2b* is expressed in the posterior midbrain and anterior hindbrain. At E13.5 and E14.5, *Phox2b* expression overlaps with the dorsal-caudal *Th* domain. (**s–w**) In coronal E14.5 sections, *Phox2b* expression overlaps with the caudal mdDA neuronal field. (**u'–w'**) *Phox2b* positive cells in the caudal midbrain co-express TH protein. *C, caudal; R, rostral; for a schematic picture of the embryonic midbrain, see figure* *1kk*.

### In situ hybridization

Embryos were collected in ice-cold buffer and immediately frozen on dry ice. Sagittal and coronal sections (14 or 16 um) were cut and collected on SuperFrost plus slides (Menzel-Glaser). In situ hybridization (ISH) with digoxigenin (DIG)-labeled RNA probes was performed as described previously [16], [17]. The following DIG-labeled probes were used: *Th*, a 1142 bp fragment of rat cDNA [18]; *Lmx1b*, a 1.3 kbp fragment containing full *Lmx1b* mouse coding sequence; *Phox2b*, a 1.6 kbp fragment containing full length coding sequence (a kind gift of J.F. Brunet).

**Figure 3:**
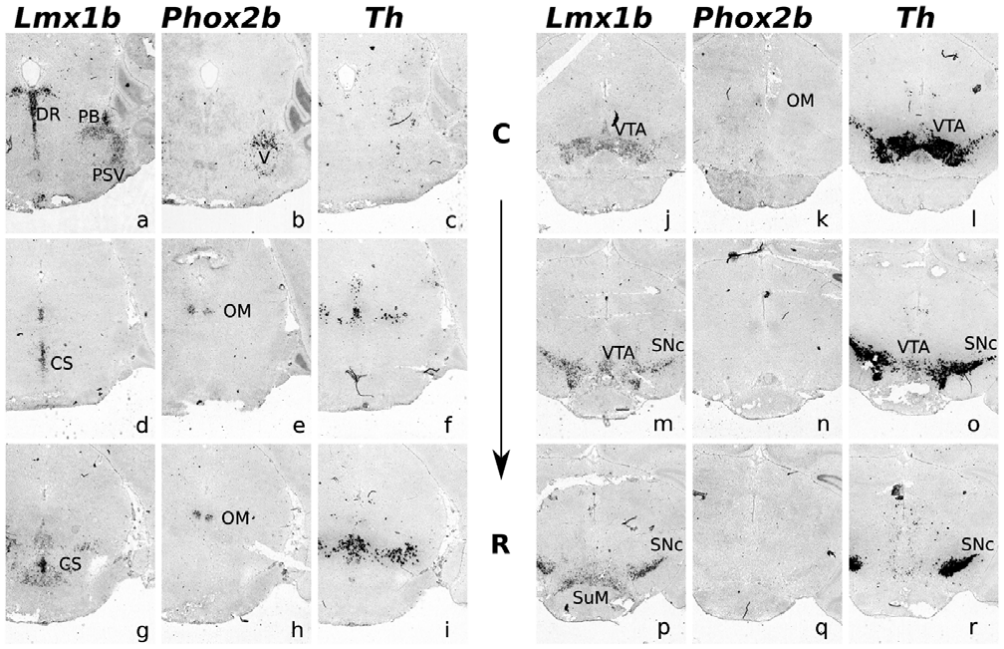
*Phox2b* transcript is absent in mdDA neurons at P14. ISH analysis of *Lmx1b, Th* and *Phox2b* in postnatal day 14 (P14) coronal mid- and hindbrain tissue. (**a–p**) *Lmx1b* expression in the hindbrain (pons) in the dorsal raphe nucleus (DR), the parabrachial nucleus (PB) and the principal sensory trigeminal nucleus (PSV). In the anterior hindbrain, *Lmx1b* is expressed in the superior central raphe nucleus (CS), and more rostrally, in the posterior midbrain, in mdDA neurons of the ventral tegmental area (VTA). Most rostrally, in the anterior midbrain, expression is observed in mdDA neurons of the substantia nigra pars compacta (SNc) and in the supramammillary nucleus (SuM). (**b–q**) In the pons, *Phox2b* is expressed in the motor nucleus of the trigeminal nerve (V), which is in the same domain as the PB and PSV but represents a different set of neurons. In the anterior hindbrain and posterior midbrain, *Phox2b* is expressed in neurons of the trochlear and oculomotor nuclei (OM). *Phox2b* is not expressed in neurons of the VTA or SNc. (**c–r**) *Th* is expressed in the periaqueductal gray (F), the retrorubral field (I) and the VTA and SNc. Some *Th* positive cells are located near the OM nuclei, but not in the same domain (I,L). *C, caudal; R, rostral*.

### Combined ISH-immunohistochemistry

ISH on fresh frozen sections was performed as described [16], [17]. After termination of the alkaline phosphatase coloring reaction of the ISH, slides were washed in 1x TBS, incubated in 0.3% H2O2 in 1x TBS for 30 min, washed again, blocked with 4% FCS in 1x TBS for 30 min, washed again and incubated overnight at 4°C with rabbit anti-TH (Pel-Freez, Arkansas, 1∶1000) in TBST (0.05 M Tris-HCl pH 7.4, 0.9% NaCl, 0.5% Triton. Next day, the sections were washed in 1x TBS, incubated for 1h with avidin-biotin-peroxidase reagent mix (ABC Elite kit, Vector Laboratories) in TBST. After this, slides were washed again, and stained with 3,3′-diamino-benzidine (DAB) until a maximum of 10 minutes. Color reaction was stopped by washing with water, slides were dehydrated with ethanol and mounted with Entellan (Merck).

**Figure 4:**
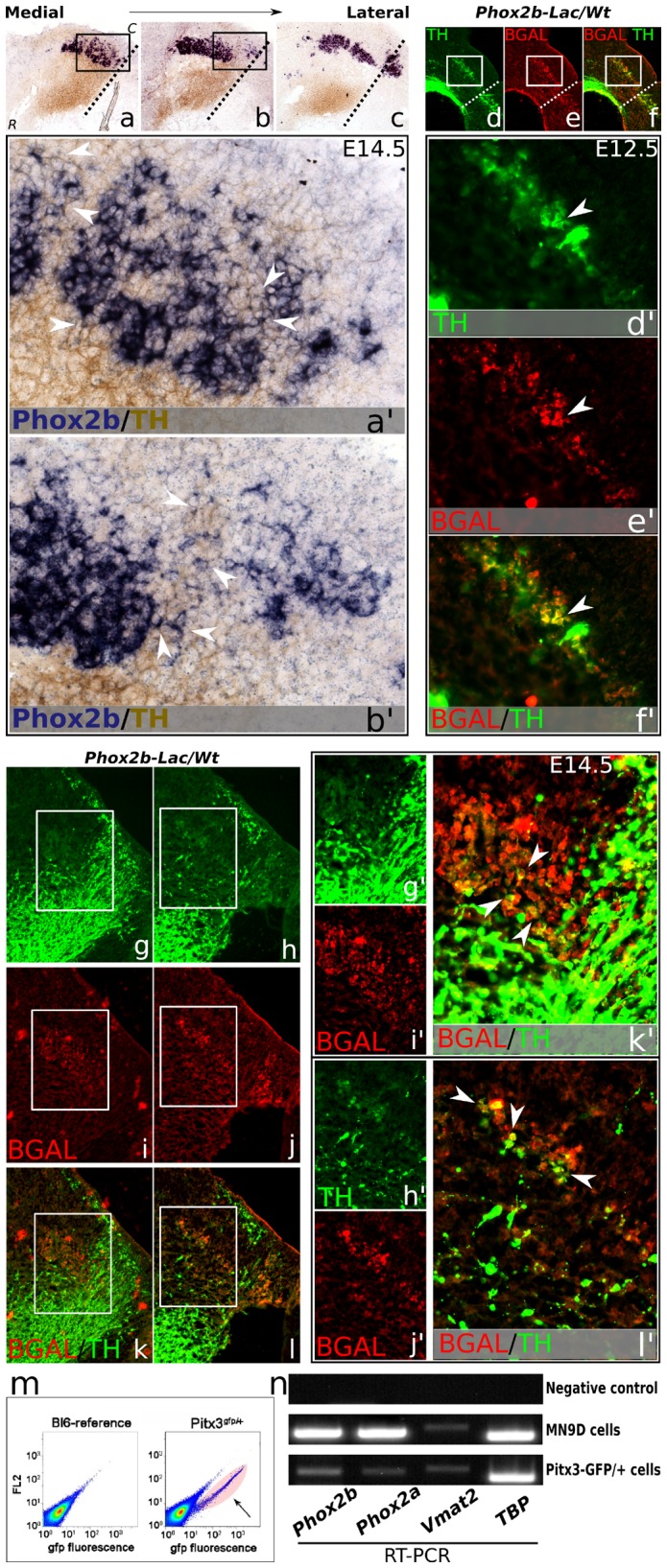
*Phox2b* is present in TH positive and *Pitx3* FAC-sorted neurons. (**a–c**) Sagittal sections of E14.5 wild-type mouse brain, from medial to lateral. TH protein staining is shown as a marker for the mdDA neuronal field (brown staining). Dashed lines represent the mid-hindbrain boundary. *Phox2b* mRNA (purple staining) is selectively expressed in the caudal midbrain and rostral hindbrain. (**a'–b'**) Medially, in the dorsal-caudal mdDA neuronal field, most *Phox2b* positive cells co-express TH protein (arrowheads). (**d–f**) E12.5 *Phox2b* heterozygous *LacZ* mutant mouse midbrain sections (*Phox2b-LacZ/Wt*) showing TH and bGAL protein co-expression in the dorsal-caudal midbrain. Dashed white lines represent the mid-hindbrain boundary. (**d'–f'**) Higher magnifications showing co-expression of TH and bGAL in the same cell (arrowhead). (**g–l**) E14.5 *Phox2b-LacZ/Wt* midbrain sections showing TH (g–h), and bGAL expression (i–j), and co-expression of both proteins according to overlay images (k–l). (**g'–l'**) Higher magnifications demonstrate that TH co-localizes with bGAL in the dorsal-caudal midbrain, confirming the in situ hybridization data (a–c). (**m**) A scatterplot showing the distribution of GFP-positive *Pitx3*-GFP/+ neurons FAC-sorted from micro-dissected E14.5 mouse midbrains, and compared with wild-type reference tissue. Only GFP-positive (pink cloud) neurons were used for mRNA isolation. (**n**) One-step RT-PCR for *Phox2a, Phox2b, Vmat2*, and *Tbp* as RT-PCR control, on RNA isolated from MN9D cells, and FAC-sorted *Pitx3*-GFP/+ neurons, confirming the presence of *Phox2b*, and *Phox2a* transcripts in mdDA neurons. *C, caudal; R, rostral; Wt, wild-type; for an embryonic midbrain reference picture, see figure* *1kk*.

### Immunohistochemistry

Embryos were directly after isolation incubated in 4% para-formaldehyde (PFA) in 1x PBS at 4°C for at least 3 h or overnight, followed by cryoprotection in 30% sucrose solution in 1x PBS. After this, embryos were frozen on dry ice. For immunohistochemistry (IHC), sections were washed twice for 5 min in 1x TBS, blocked in 4% fetal calf serum (FCS) in 1x TBS for 30 min, and were washed again. When incubating with sheep anti-TH, blocking was performed in 5% normal donkey serum in 1x TBS. Slides were incubated with primary antibody in THZT (50 mM Tris-HCl pH 7.6, 0.5 M NaCl, 0.5% Triton) at 4°C overnight, washed 3x with 1x TBS for 5 min and incubated for 1 h with secondary antibody in THZT at room temperature. Slides were washed three times in 1x PBS for 10 min and mounted using FluorSave (Calbiochem, Darmstadt). Antibodies used: rabbit anti-TH (Pel-Freez, Arkansas, 1∶1000), sheep anti-TH (Millipore, 1∶500), mouse anti-b-GAL (Promega, 1∶300), rabbit anti-b-GAL (Cappel, 1∶1000). Secondary antibodies: goat anti-rabbit Alexa-Fluor-488, donkey anti-sheep Alexa-Fluor-488, goat anti-mouse Alexa-Fluor-555, goat anti-rabbit Alexa-Fluor-555, all 1∶1000 (Invitrogen).

**Figure 5:**
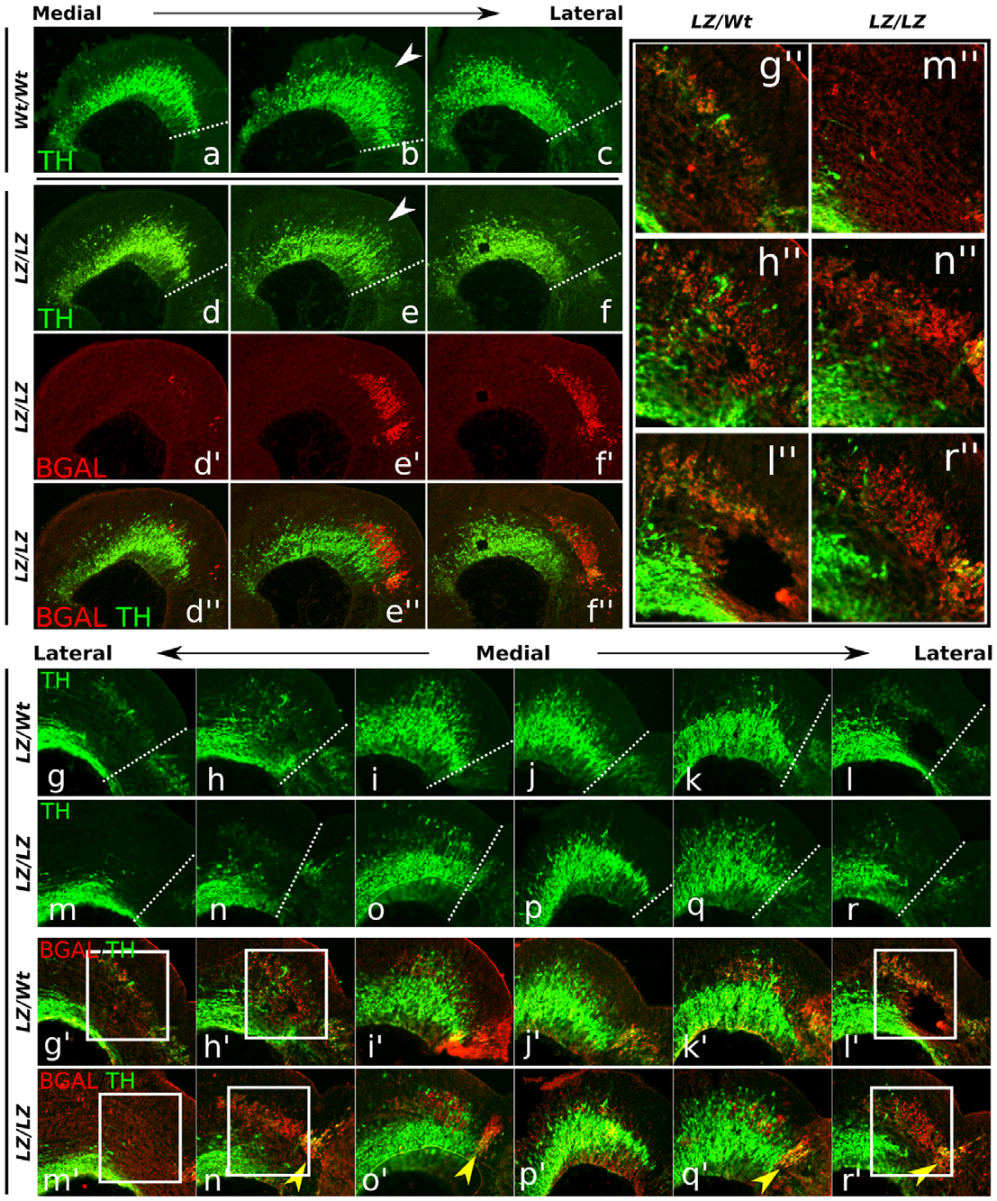
Loss of TH expression in E12.5 *Phox2b-LacZ/LacZ* embryonic brains. (**a–f**) TH protein expression in E12.5 *Phox2b* wild-type and homozygous mutant (*LZ/LZ*) littermates. Mild loss of TH is observed in the dorsal-caudal midbrain (arrowhead). Dashed white lines represent the mid-hindbrain boundary. (**d'–f'**) bGAL expression in cells normally expressing *Phox2b*, rostrally and caudally of the MHB, plus overlays with TH (**d''–f''**). (**g–l**) TH expression in the heterozygous *Phox2b-LacZ* mutant (*LZ/Wt*), compared to homozygous *Phox2b* mutant (*LZ/LZ*) littermates (**m–r**). (**g'–l'**) Co-expression of bGAL and TH is shown in the heterozygous mutant, whereas TH expression is lower in bGAL positive cells in the homozygous mutant (**m'–r'**). (**g''–r''**) bGAL expressing neurons also express TH. In *Phox2b* mutant neurons (bGAL positive cells), the number of cells co-expressing TH protein appears lower, as demonstrated by decreased co-localization (m'',n'',r''). *LZ, Phox2b-LacZ mutant; Wt, wild-type; for an embryonic midbrain reference picture, see figure* *1kk*.

**Figure 6:**
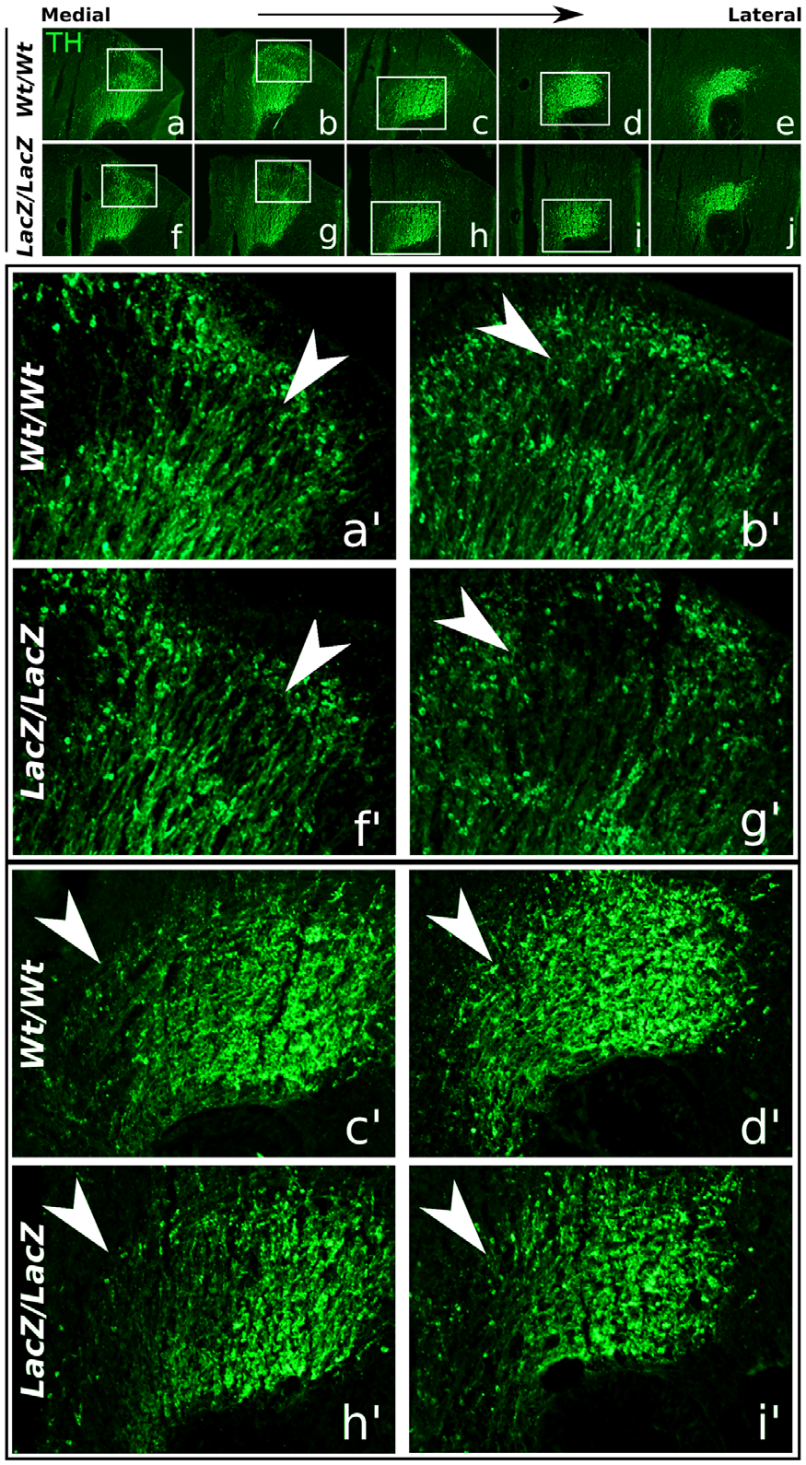
TH expression in E14.5 *Phox2b-LacZ/LacZ* embryonic brains compared to wild-type littermates. (**a–j**) TH protein expression in wild-type (*Wt/Wt*) and *Phox2b* mutant (*LacZ/LacZ*) littermates. Medially, the dorsal-caudal area where *Phox2b* normally is expressed, shows a decrease or loss of TH expression in the *Phox2b* mutant (boxed areas and (**a'–b',f'–g'**)). In addition, a subtle decrease of TH expressing neurons is shown in the rostral domain of the mdDA system (**c'–d',h'–i'**). *LacZ, Phox2b-LacZ mutant; Wt, wild-type; for an embryonic midbrain reference picture, see figure* *1kk*.

### Fluorescence-activated cell sorting (FACS)

The micro-dissected mdDA region of several *Pitx3-GFP/+* embryos was dissociated using a Papain dissociation system (Worthington) and cells were sorted on a Cytopeia Influx Cell sorter. Sort gates were set on forward scatter versus side scatter (life cell gate), on forward scatter versus pulse width (elimination of clumps) and on forward scatter versus fluorescence channel 1 (528/38 filter; GFP fluorescence). Cells were sorted (98% purity) using a 100 um nozzle at a pressure of 15 PSI with an average speed of 7000 cells/second and collected in Trizol reagent (Invitrogen). [15].

### One-step RT PCR

Total RNA was purified from MN9D cells [19]or *Pitx3-GFP/+* FAC-sorted neurons, using Trizol according to manufacturer's protocol (Invitrogen). Gene expression levels were determined using a One-step RT-PCR kit (Qiagen). We used 1 ng total RNA from MN9D per 20 uL PCR reaction. Samples were separated on 1.5–2.0% agarose gels, and gels were scanned using a FLA-5000 imaging system (Fuji). Primers used: *Phox2a* Forward primer 5′-GCTTTCTTAGGAACAGGGATC, *Phox2a* Reverse primer 5′-GGCTCTTCCCCTCTAGTGTC (product size 228 bp); *Phox2b* Forward primer 5′-CAAAGAGTTGGAGAGGGTC, *Phox2b* Reverse primer 5′-CTTTGCTCTCGTCGTCC (product size 226 bp); *Slc18a2* (*Vmat2*) Forward primer 5′-GCTATGCCTTCCTGCTGATC, Vmat2 reverse primer 5′-AGCTGAATAGCTCCAATCCAAG (product size 259 bp).

## Results

### *Phox2b* is expressed in developing mdDA neurons

Since *Phox2a* and *Phox2b* are paralogues and it is suggested that *Phox2b* and *Lmx1b* can regulate *Phox2a* [4], [12], [13], we were interested whether *Phox2b* is expressed in a similar, specific pattern in the dorsal-caudal mdDA neuronal field as we previously found for *Phox2a* (unpublished data). Therefore, we analyzed the expression pattern of *Phox2b* by means of in situ hybridization (ISH) on E12.5, E13.5 and E14.5 sagittal sections of wild-type (C57Bl/6J) embryos, and compared this to *Lmx1b* expression (Fig. 1).

At E12.5, *Phox2b* was clearly expressed in the posterior midbrain, and anterior hindbrain (Fig. 1G–L), except for the most medial sections, where no expression was detected (Fig. 1i). In the lateral areas, where *Phox2b* was highly expressed, this expression overlapped with the caudal domain of *Lmx1b*. At E13.5 and E14.5, *Phox2b* was also expressed in the most medial sections (Fig. 1S–X,EE–JJ). When comparing with *Lmx1b* expression, in both developmental stages a clear overlap of both *Phox2b* and *Lmx1b* expression fields was observed. In order to asses the possibility that *Phox2b* is involved in developing mdDA neurons, we compared *Phox2b* expression to *Th*. At E12.5, *Phox2b* only partially overlapped with *Th* (Fig. 2A,D), whereas more laterally, *Phox2b* was expressed more caudally and outside the *Th* domain (Fig. 2B,C,e,F). At E13.5, major overlap of *Phox2b* with the dorsal-caudal *Th* domain was observed, a pattern that was confirmed at E14.5 (Fig. 2g–r). In addition, by using *Phox2b* ISH analysis together with TH protein staining, the observed dorsal-caudal overlap was further validated in coronal brain sections, where many TH positive neurons overlapped with *Phox2b* expression, mainly in the dorsal-caudal mdDA domain (Fig. 2s–w').

In summary, *Phox2b* is expressed in the caudal midbrain, at high levels during early and late developmental stages. At E13.5 and E14.5 it largely overlaps with dorsal-caudal *Lmx1b* and *Th* midbrain expression domains, suggesting a possible role during the development of this mdDA neuronal subset.

### *Phox2b* expression is restricted to the developing mdDA neuronal field

The expression of *Phox2b* in the developing midbrain may relate to the known role of *Phox2b* in the development of (midbrain) oculomotor neurons (OMNs). However, it might also suggest an additional involvement in the development of mdDA neurons. In the adult mouse brain, *Phox2b* is expressed in the hindbrain in branchiomotor and visceromotor neurons [6], [11], [20]. In addition, it is expressed in the noradrenergic system (locus ceruleus and lateral tegmental area) and in oculomotor and trochlear neurons [10], [21]–[25]. Here we investigated the postnatal *Phox2b* midbrain expression, to determine whether *Phox2b* is present in mature mdDA neurons.

Therefore, we performed *Phox2b*, *Lmx1b* and *Th* ISH analysis on P14 coronal sections of wild-type mice (Fig. 3). *Lmx1b* expression has been analyzed before [26], [27], and we confirmed *Lmx1b* expression in the (dorsal) raphe nucleus (DR), the parabrachial nuclei (PB) and principal sensory trigeminal nucleus (PSV) (Fig. 3a). In adjacent sections, no *Phox2b* expression was observed in these domains. However, close to the expression domains of *Lmx1b* in the PB and PSV, *Phox2b* was expressed in neurons of the motor nucleus of the trigeminal nerve (V) (Fig. 3b). More rostrally in the anterior hindbrain and posterior midbrain, *Phox2b* expression was confirmed in the oculomotor nuclei (OM) (Fig. 3E,H,K). Importantly, no *Lmx1b* expression or *Th* expression was observed in the OM (Fig. 3D,G,F,I). Clear expression of *Th* and *Lmx1b* was found in the VTA and SNc, and the latter gene was also expressed in the supramammillary nucleus (SuM) (Fig. 3l,o,r and J,M,P). However, no mdDA specific *Phox2b* expression was detected in these areas (Fig. 3K,N,Q), indicating that *Phox2b* is not expressed in mature mdDA neurons.

### *Phox2b* is restricted to a caudal subset of mdDA neurons at E14.5

To further substantiate *Phox2b* expression in developing mdDA neurons, we performed *Phox2b* ISH analysis, together with TH protein analysis, on E14.5 sagittal wild-type tissue (Fig. 4). We confirmed the specific expression of *Phox2b* in a selective TH positive domain of the dorsal-caudal midbrain (Fig. 4a–c), as was observed previously in coronal sections. Moreover, medially, many cells in this domain co-expressed *Phox2b* mRNA and TH protein (Fig. 4a'–b', arrowheads).

To investigate this co-expression into more detail, we analyzed TH and bGAL protein expression in E12.5 and E14.5 *Phox2b-LacZ* mouse midbrains [14]. Importantly, in these *LacZ* knock-in mice, we observed TH expressing neurons that clearly co-expressed bGAL (Fig. 4d–f', arrowheads). In line with this, also at E14.5, co-expression of TH and bGAL was demonstrated, in a small group of cells in the dorsal-caudal midbrain (Fig. 4g–l'). Altogether, the combined data from ISH and IHC expression analyzes, show that *Phox2b* is co-expressed in this caudal subset of mdDA neurons.

To further validate that *Phox2b* is expressed in mdDA neurons, we used isolated RNA from FAC-sorted *Pitx3-GFP/+* neurons (E14.5) (Fig. 4m), and subjected this material to one-step RT PCR. In addition, we used RNA from MN9D cells as a positive control, since *Phox2b* is expressed in this dopaminergic cell line. We analyzed transcript levels of *Phox2b*, *Phox2a* and *Vmat2*, the latter as a positive control. *Tbp* was taken along as a loading and PCR reference. We confirmed expression of all transcripts in MN9D cells, where the two *Phox2*-genes were highly expressed, when compared to *Vmat2* (Fig. 4n). Importantly, also in *Pitx3-GFP/+* neurons, *Phox2b* transcript was present, in comparable levels as *Vmat2*.

In conclusion, by using several approaches, we showed that *Phox2b* is present in a dorsal-caudal subset of developing mdDA neurons.

### Caudal TH expression is affected in *Phox2b* mutants

The expression of *Phox2b* in mdDA neurons implicates a role in mdDA neuronal development. To investigate this into more detail, we analyzed TH expression in the developing midbrain of *Phox2b* null mutant embryos.

Analysis of E12.5 wild-type and *Phox2b-LacZ/LacZ* (knock-out/knock-in) tissue (Fig. 5a–f) revealed a subtle decrease in TH expression in the most dorsal-caudal TH expression domain of the mdDA neuronal field (Fig. 5e, arrowhead). Within this region, b-GAL showed a high expression level (Fig. 5d'–f''). Furthermore, when comparing *Phox2b* heterozygous with *Phox2b-LacZ/LacZ* embryonic brains, the restricted loss of dorsal-caudal TH expression was confirmed (Fig. 5, compare g–l with m–r). In the heterozygous dorsal-caudal mdDA neuronal field, a select group of cells was observed, that clearly co-expressed TH and bGAL (Fig. 5g'–l'). In the absence of *Phox2b*, this subset displayed lower levels of TH, as was shown by decreased co-localization of b-GAL with TH (Fig. 5m'–r'). This loss appeared to be midbrain specific, since a small group located directly posterior of the MHB clearly still co-expressed TH and b-GAL in *Phox2b-LacZ/LacZ* embryos (Fig. 5n,o,q,r, arrowheads). In conclusion, the loss of *Phox2b* results in a decreased TH expression in a caudal subset of mdDA neurons specifically (Fig. 5g''–r'').

Similar results were observed in the E14.5 *Phox2b* null mutant. A subtle reduction of TH positive neurons was observed in the *Phox2b* area in the medial midbrain (Fig. 6a–b',f–g', arrowheads). Interestingly, another mild deficit was observed, in an area that does not express *Phox2b* at this stage at all. The entire mdDA neuronal field, from lateral to medial, displayed a subtle reduction in TH expression in the rostral (diencephalic) mdDA neuronal field (Fig. 6c–d',h–i', arrowheads).

Taken together, *Phox2b* is expressed in a subset of neurons that expresses mild levels of TH, in the dorsal-caudal midbrain and in a small group of TH positive cells located near the isthmic organizer. This isthmic group is spared whereas the more anterior located TH positive cells lose TH expression as a consequence of *Phox2b* ablation, suggesting that *Phox2b* is involved in the correct specification of this small subset of mdDA neurons.

## Discussion

*Phox2a*, and its paralogue *Phox2b*, play important roles in the development of branchiomotor and visceromotor neurons in the ventral hindbrain and are both expressed in noradrenergic centers [6], [10], [11], [20], [25], [28]–[30]. In the main noradrenergic center, the locus ceruleus, both genes can fully compensate for each other [25]. In addition, both factors are also specifically expressed in midbrain oculomotor neurons and in trochlear neurons, where *Phox2a* expression precedes that of *Phox2b* [4], [6], [8], [12].

Since *Phox2a* displayed an expression pattern that was largely overlapping with the dorsal-caudal mdDA neuronal field, in an earlier study (unpublished data), we suspected a role for *Phox2a*, and *Phox2b*, in the development of these neurons, in addition to the proposed function in oculomotor neuron (OMN) development. Indeed, our current analysis of *Phox2b* provides insight in a role of this gene in the development of a subset of mdDA neurons.

### *Phox2b* is temporally expressed in a subset of developing mdDA neurons

Our detailed analysis of the expression pattern of *Phox2b* during several developmental stages, confirms that *Phox2b* is expressed in a specific pattern overlapping with the dorsal-caudal mdDA neuronal field. However, in this midbrain area, motorneurons are formed as well. Moreover, a known marker for motorneurons, *Isl1*, is expressed in this area in a similar expression pattern as *Phox2a* and *Phox2b* [8], [31]online expression databases). The *Phox2* genes are involved in OM development, and since a recent paper suggested a role for *Phox2a* together with *Lmx1b* in the generation and control of OMNs and red nucleus neurons (RNNs) [4], it is likely that *Phox2a* and *Phox2b* expressing cells in the midbrain are (immature) visceral and somatic motorneurons.

In this study, we aimed to investigate in detail whether *Phox2b* is truly confined to OMNs, or in addition plays a role in mdDA neuronal development. Intriguingly, we clearly showed that many *Phox2b* expressing cells in the midbrain area, co-express TH protein, which was confirmed in *Phox2b-LacZ* positive neurons, and by RT-PCR on FAC-sorted *Pitx3*-positive neurons. In contrast to this, analysis of postnatal mouse brains revealed that *Phox2b* is not expressed in mature mdDA neurons. We confirmed expression in known sites of *Phox2b*, in the ventral hindbrain, and in the OM. However, no expression rostral to this nucleus was observed. Since *Phox2b* is clearly expressed in the developing mdDA neuronal field, the lack of expression in the postnatal mdDA system indicates a role for *Phox2b* in a small but specific subset of the mdDA domain during development of these neurons.

### *Phox2b* is involved in the specification of a small caudal subset of mdDA neurons

In line with the expression of *Phox2b* in a dorsal-caudal subset of developing mdDA neurons, a loss of TH was observed in the homozygous *Phox2b* mutant, in this specific domain. Furthermore, a small decrease in the rostral expression domain (diencephalon) of TH was identified, suggesting that impaired expression in the dorsal caudal part of the developing mdDA neuronal field, might additionally influence a rostral subset of neurons. This may be a consequence of failure of migration of neurons from the medial-caudal region, or represent a more general, non-cell autonomous defect.

To conclude, our data not only identified *Phox2b* temporal expression in a select group of developing mdDA neurons, but also revealed a role for this gene in the development of these neurons. Thus, besides the known role of both *Phox2*-genes in OM development, our data suggest that *Phox2b* is involved in the correct specification of a small caudal subset of mdDA neurons.
